# Perianal Paget’s disease: a clinicopathological and immunohistochemical study of 13 cases

**DOI:** 10.1186/s13000-020-00952-w

**Published:** 2020-03-24

**Authors:** Xiaoyan Liao, Xiuli Liu, Xuemo Fan, Jinping Lai, Dongwei Zhang

**Affiliations:** 1 Department of Pathology and Laboratory Medicine, University of Rochester Medical Center, Rochester, NY USA; 2 Department of Pathology, Immunology and Laboratory Medicine, University of Florida College of Medicine, Gainesville, FL USA; 3 Department of Pathology and Laboratory Medicine, Cedars-Sinai Medical Center, Los Angeles, CA USA; 4 Department of Pathology and Laboratory of Medicine, Kaiser Permanente Sacramento Medical Center, Sacramento, CA USA

**Keywords:** Paget’s disease, Perianal, Clinicopathologic features, Histology, Immunohistochemistry, Prognosis

## Abstract

**Background:**

Perianal Paget’s disease (PPD) is rare and mostly described in clinical literature as case reports or small series.

**Methods:**

We investigated the clinicopathologic and immunohistochemical features of PPD in a total of 13 cases retrieved from multiple academic institutions.

**Results:**

The median age at diagnosis was 75 (range 50–86) years. Males were predominant with a male to female ratio of 2.25:1. Four (30.8%) cases were classified as primary PPD due to lack of synchronous or metachronous underlying malignancies, while nine (69.2%) were classified as secondary PPD with concurrent invasive adenocarcinoma (*n* = 8) or tubular adenoma with high-grade dysplasia (*n* = 1). Immunohistochemically, there is no differential expression of CK7 or CK20 in Paget’s cells between primary and secondary PPD; however, GCDFP-15 was only positive in primary PPD (3/3 vs. 0/6, *P* = 0.012), while CDX2 was only positive in secondary PPD (0/3 vs. 7/7, *P* = 0.008), suggesting different cell origin. All patients received local surgical resection with or without adjuvant therapy. After a median follow-up of 47 months, one patient with secondary PPD (7.7%) died of disease progression from underlying adenocarcinoma.

**Conclusions:**

PPD occurs in elderly patients with male predominance and is frequently associated with underlying malignancies. Differential expression of CDX2 and GCDFP-15 may help distinguishing primary vs. secondary PPD, which is important for management as the presence of an underlying malignancy impacts clinical course and prognosis. Surgical excision remains the major treatment strategy for PPD. Long-term follow-up is required to monitor the disease recurrence and metastasis.

## Introduction

Extramammary Paget’s disease is a rare neoplastic condition of apocrine gland-bearing regions [1, 2]. The most frequently affected site is vulva, followed by perineal, perianal, scrotal and penile skin. Perianal Paget’s disease (PPD) involving perianal skin or anal mucosa accounts for less than 20% of extramammary Paget’s disease. Primary PPD is very rare. It is an indolent disease, but can recur with a recurrence rate of 44–60% [3, 4]. Up to 60% of PPD were associated with underlying malignancies [5, 6], in which the Paget’s cells represent intraepithelial spread of an existing dermal adnexal or visceral adenocarcinoma [7, 8]. Thus, the findings of PPD should prompt diligent search for an underlying malignancy [9–12]. The new WHO book classifies anal adenocarcinomas as primary if arising from mucosal glandular epithelium, which shares the same immunoprofile as colorectal adenocarcinoma (CK7+/−, CK20+/CDX2+), or from anal glands, which shares the same immunoprofile as skin adnexal carcinoma (CK7+/CK20−/CDX2-) [13]. Thus, proper diagnosis relies not only on immunoprofile, but also clinical information and macroscopic tumor location [14, 15]. PPD has been rarely described in literatures as single case report or small case series [9, 16–18]. Yet, much is still unknown due to its rarity. The main goal of this study is to conduct a multi-institutional study to investigate the clinical, histomorphological, immunohistochemical, and molecular genetic features of PPD.

## Materials and methods

### Patients

Thirteen patients with PPD were identified between 1999 and 2019 from three large medical centers (University of Rochester Medical Center, University of Florida College of Medicine, and Cedars-Sinai Medical Center) in the United States. Clinical data including patient demographics, medical history, presenting symptoms, physical examination, treatment, and outcome were collected through electronic medical record.

### Histomorphological evaluation and immunohistochemistry

Hematoxylin and eosin (H&E)-stained slides from all cases were reviewed by two experienced gastrointestinal pathologists to confirm diagnosis and for histopathologic analysis. Immunohistochemistry was performed on 4-μm-thick slides prepared from formalin fixed, paraffin embedded tissue blocks on an automated immunostainer (Ventana BenchMark Ultra) according to the manufacturer’s instructions. Heat induced epitope retrieval (HIER) was performed for antigen retrieval. Appropriate controls were used throughout. The following antibodies were used: cytokeratin 7 (CK7, Cat# GA619, Agilent, Santa Clara, CA), cytokeratin 20 (CK20, Cat# GA777, Agilent, Santa Clara, CA), CDX2 (Cat# DAK-CDX2, Agilent, Santa Clara, CA), carcinoembryonic antigen (CEA, Cat# GA622, Agilent, Santa Clara, CA), MUC1 (Cat# CM319B, Biocare Medical, Pacheco, CA), MUC2 (Cat# PA0155, Leica, Buffalo Grove, IL), GCDFP-15 (Cat# GA077, Agilent, Santa Clara, CA), GATA3 (Cat# CM405B, Biocare Medical, Pacheco, CA), and p40 (Cat# ACI3066, Biocare Medical, Pacheco, CA). Fisher’s exact test was used to compare frequencies between two groups (primary vs. secondary PPD). A *P*-value of < 0.05 was considered statistically significant.

## Results

### Clinical characteristics

The clinical characteristics of all 13 cases were summarized in Table 1. The median age at diagnosis was 75 (range 50–86) years. Males were predominant with a male to female ratio of 2.25:1. Clinical presentations included itching and irritation of the perianal area. Physical examination often showed erythema, plaques, ulceration, fissure, fistula, or mass lesions. Four (4/13, 30.8%) patients presented as primary PPD with no synchronous or metachronous underlying anorectal malignancies, while nine (9/13, 69.2%) were classified as secondary PPD due to concurrent invasive adenocarcinoma (*n* = 8) or tubular adenoma with high-grade dysplasia (*n* = 1). In addition, five patients had other malignancies, including basal cell carcinoma, breast carcinoma, urothelial carcinoma in-situ, chronic myeloid leukemia, and follicular lymphoma.

**Table 1:** Demographics and clinical features of 13 patients with perianal Paget’s disease

Case	Age (years)	Sex	Clinical presentation	Other malignancies	Underlying malignancy (Immunoprofile)	Metastasis	Outcome	Follow up (month)	Treatment other than surgery
1	75	M	Perianal erosive plaque	Chronic myeloid leukemia	None	N/A	Died^a^	60	Radiation
2	79	F	Discomfort	Follicular lymphoma; breast carcinoma; urothelial carcinoma in-situ	None	N/A	Alive	83	None
3	61	M	Itching and irritation of the perianal area; erythema with whitish plaques	None	None	N/A	Alive	31	None
4	86	M	Perianal lesion	Basal cell carcinoma	None	N/A	Alive	4	
5	81	M	Discomfort	None	Mixed moderately differentiated adenocarcinoma with mucinous features and neuroendocrine (large cell) carcinoma (CK7+/CK20+/CDX2+)	Lymph nodes and liver	Died^a^	79	None
6	53	F	Acute anal fissure, anal lesion	Basal cell carcinoma; breast carcinoma	Adenocarcinoma, well differentiated (CK7−/CK20+/CDX2+)	None	Alive	61	None
7	86	M	Discomfort	None	Adenocarcinoma, poorly differentiated (CK7+/CK20-)	None	Alive	52	None
8	69	F	None (detected during routine colon cancer screening biopsy)	None	Adenocarcinoma, moderately differentiated (CK7+/CK20+/CDX2+)	None	Alive	16	None
9	83	M	Perianal rash and lesion with ulceration	Basal cell carcinoma	Adenocarcinoma, moderately differentiated (CK7−/CK20+/CDX2+)	None	Alive	7	Chemoradiation
10	69	F	Anal/perianal rash and lesion	None	Adenocarcinoma with neuroendocrine and signet ring features (CK7+/CK20+/CDX2+)	None	Alive	21	None
11	50	M	Anal fistula	None	Adenocarcinoma with mucinous features	Lymph nodes	Alive	227	Chemotherapy
12	70	M	Perianal lesion	None	Mucinous adenocarcinoma (CK7+/CK20+/CDX2+)	Lymph nodes, liver and bone	Alive	47	Chemoradiation
13	77	M	None	None	Tubular adenoma with high-grade dysplasia (CK7−/CK20+/CDX2+)	None	Alive	6	None

^a^Case #1 died of chronic myeloid leukemia. Case #5 died of disease progression. N/A, not applicable

### Pathologic findings

**Fig. 1:**
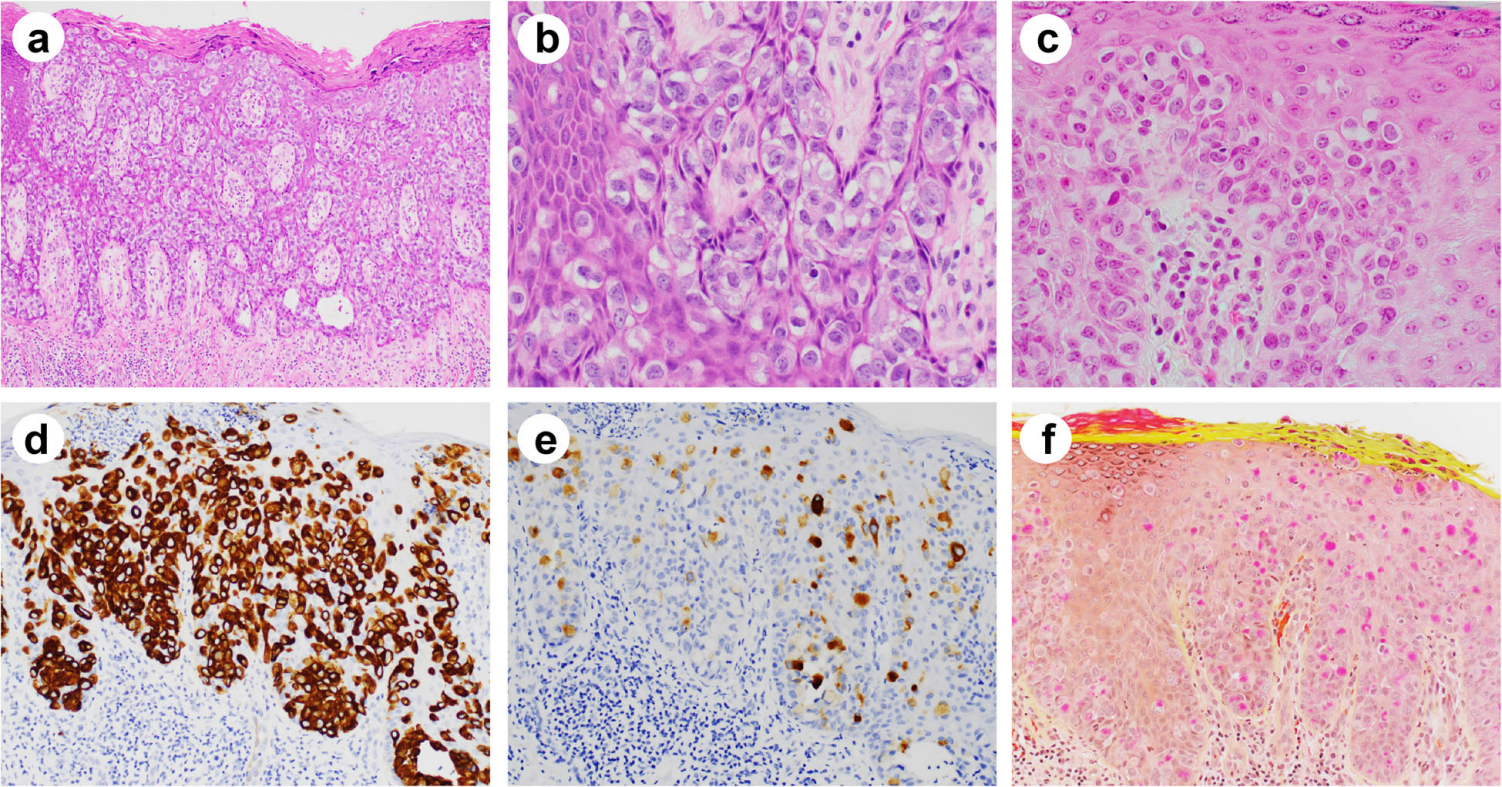
Histologic features and special stains of Paget’s cells in primary perianal Paget’s disease. **a**, **b** Paget’s cells in primary perianal Paget’s disease are large, hyperchromatic with pale cytoplasm and prominent nucleoli (hematoxylin and eosin; a, × 100; b, × 400). The Paget’s cells in primary perianal Paget’s disease (**c**, hematoxylin and eosin) are positive for CK7 (**d**) and GCDFP-15 (**e**) by immunohistochemistry. **f** Mucicarmine stain highlights intracellular mucin. Magnifications: c-f, × 200

**Fig. 2:**
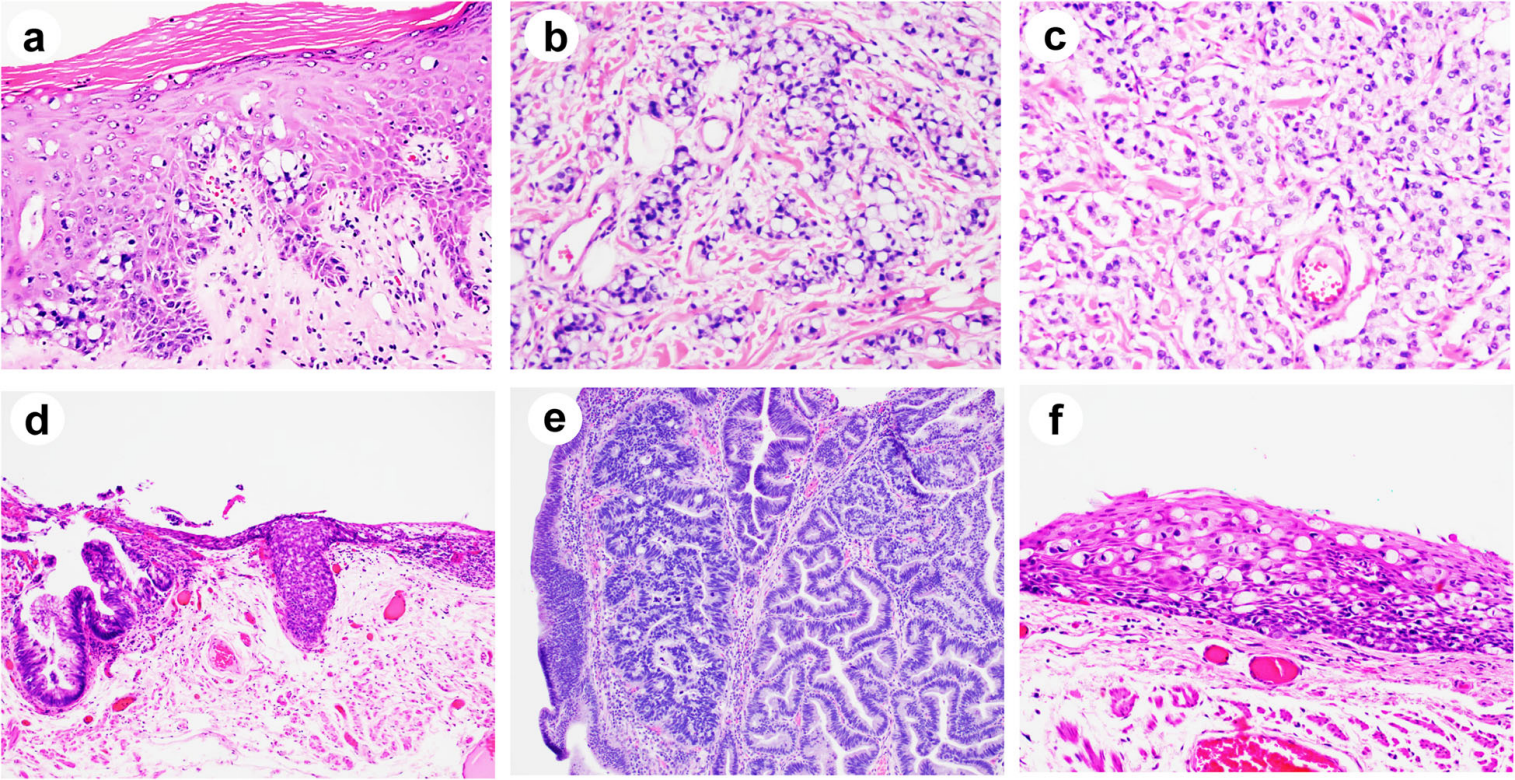
Histologic features of secondary perianal Paget’s disease cases associated with underlying malignancy (hematoxylin and eosin). **a-c** High magnification (× 200) showed intraepithelial Paget’s cells (**a**) and underlying invasive adenocarcinoma with signet ring cell feature (**b**) and neuroendocrine feature (**c**). **d** Low magnification (× 100) showed intraepithelial Paget’s cells associated with a tubular adenoma with high-grade dysplasia. Higher magnification views (× 200) of (d) showed tubular adenoma with high-grade dysplasia (**e**) and Paget’s cells (**f**)

**Fig. 3:**
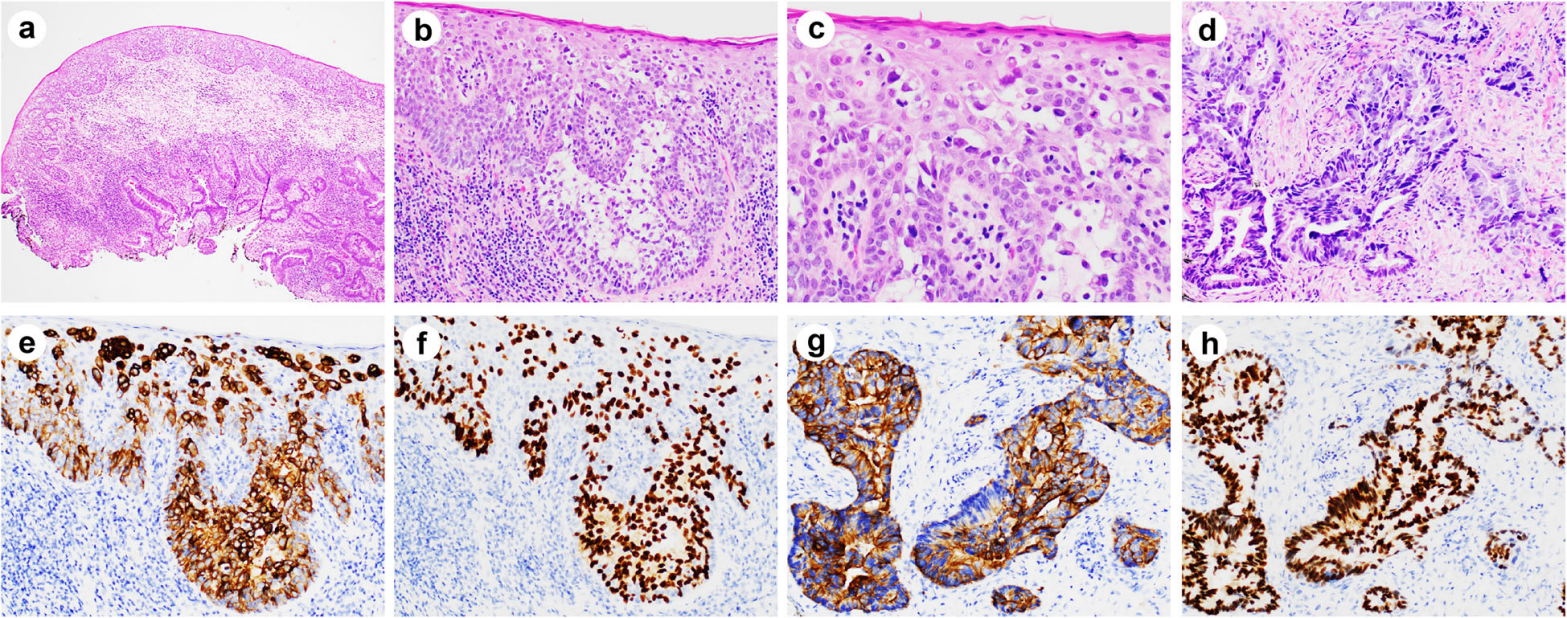
Histologic and immunohistochemical features of secondary perianal Paget’s disease with associated invasive adenocarcinoma. **a** Low magnification showed intraepithelial Paget’s cells and underlying conventional invasive adenocarcinoma. **b-d** High magnification view of (**a**) showed Paget’s cells (**b**, **c**) and invasive adenocarcinoma (**d**). Paget’s cells are positive for CK20 (**e**) and CDX2 (**f**) by immunohistochemistry. Underlying adenocarcinoma cells are also positive for CK20 (**g**) and CDX2 (**h**) by immunohistochemistry. Magnifications: a, × 40; b, × 200; c, × 400; d-h, × 200

Histologically, all PPD cases showed intraepithelial infiltration by sheets and clusters of large atypical neoplastic cells. The intraepidermal Paget’s cells were large, hyperchromatic and pleomorphic with clear or pale cytoplasm, and occasionally prominent nucleoli. For cases classified as primary, the Paget’s cells were mostly singly dispersed, with occasional visible intracytoplasmic mucin and rare glandular formation (Fig. 1a-c). In contrast, for cases classified as secondary, the Paget’s cells were more mucinous, frequently with eccentric nuclei and signet ring cell appearance, resembling the underlying carcinomatous cells (Figs. 2 and 3). Interestingly, mucinous differentiation was noted in 3 of 8 underlying adenocarcinomas. In one case where only tubular adenoma with high-grade dysplasia was identified, the Paget’s cells were also mucinous with signet ring like appearance, resembling some of the high-grade dysplastic cells (Fig. 2d, e). Of note, two underlying adenocarcinomas had neuroendocrine differentiation, among which one showed both mucinous and neuroendocrine components (Fig. 2a-c).

### Immunohistochemical profile

Immunohistochemical studies were performed in all cases except one (Table 2). The Paget’s cells were frequently positive for CK7 (9/12; 75%), CK20 (9/12; 75%), CDX2 (7/10; 70%), CEA (6/7; 85.7%), MUC1 (5/8; 62.5%), MUC2 (8/8; 100%), GATA3 (4/7, 57.1%), and GCDFP-15 (3/9; 33.3%) (Fig. 1d, e). Mucicarmine stain highlighted intracellular mucin in all of the cases tested (Fig. 1f). Secondary Paget’s cells shared the same immunoprofile as the invasive adenocarcinoma component, suggesting the same tumor origin (Fig. 3e-h). Interestingly, while there was no differential expression of CK7, CK20, CEA, MUC1, MUC2 between primary and secondary PPD, GCDFP-15 was only positive in primary PPD (3/3 vs. 0/6, *P* = 0.012), while CDX2 was only positive in secondary PPD (0/3 vs. 7/7, *P* = 0.008). GATA3 was positive in all primary PPD (3/3, 100%), and 1 of 4 secondary PPD (1/4, 25%) tested. All cases were negative for squamous cell markers such as p40 or p63. SOX10, MelanA, p53, p16, HER2, PAX8 were also tested in some of the cases and were all negative (data not shown). Immunoprofiling of 8 cases with underlying malignancies (Table 1) indicated that only one case (case #7) is likely anal gland origin (CK7+/CK20-), while the others are likely colorectal primary (CK20+/CDX2+).

**Table 2:** Immunohistochemical profile of perianal Paget’s disease

	Primary				Secondary									*P* value
	1	2	3	4	5	6	7	8	9	10	11	12	13	
Cytokeratin 7	+	+	+	+	+	–	+	+ (F)	–	+	ND	+	–	NS
Cytokeratin 20	–	–	+	+ (F)	+	+	–	+	+	+	ND	+	+	NS
CDX2	–	–	ND	–	+	+	ND	+	+	+	ND	+	+	0.008
MUC1	+	+	ND	+	+	–	ND	–	ND	ND	ND	+	–	NS
MUC2	+	+	ND	+ (F)	+	+	ND	+	ND	ND	ND	+	+	NS
CEA	+	–	ND	+ (F)	ND	+	+	+	ND	+	ND	ND	+	NS
GCDFP-15	+ (F)	+	ND	+ (F)	–	–	ND	–	ND	–	ND	–	–	0.012
P40	–	–	ND	–	–	–	ND	–	ND	ND	ND	ND	–	NS
Mucicarmine	+	+	+	+	ND	+	+	+	ND	ND	ND	ND	+	NS
GATA3	+	+	ND	+	ND	–	ND	–	ND	ND	ND	–	+	NS

(*F*) Focally positive, *ND* Not done, *NS* Not significant

Mismatch repair proteins (MMR) immunohistochemistry and/or microsatellite instability PCR were performed in three invasive adenocarcinomas and all showed to be MMR proficient. Next-generation sequencing using a 74 cancer-related gene panel was performed in one case of invasive adenocarcinoma with neuroendocrine and signet ring cell features (case #10) but did not reveal any known cancer-related genetic mutations in the panel.

### Treatment and follow-up

All patients received local/extensive surgical excision of the perianal lesions as well as underlying adenocarcinomas. Four patients, including 1 primary PPD and 3 secondary PPD, received radiation and/or chemotherapy after the surgery (Table 1). After a median follow-up of 47 (range 4–227) months, all patients with primary PPD survived except one died of other malignancy (chronic myeloid leukemia). Majority (8/9, 88.9%) of patients with secondary PPD survived, yet 2 (22.2%) patients had recurrence of underlying adenocarcinoma, 3 (33.3%) developed lymph node, liver or bone metastasis, and 1 (case #5, 11.1%) died of underlying adenocarcinoma with nodal and liver metastasis. Overall, the total mortality of PPD was 15.4% and disease-specific mortality was 7.7%.

## Discussion

In this multi-institutional study, we analyzed a series of PPD to characterize its clinicopathologic and immunophenotypic features. We found that PPD occurs in elderly patient with male predominance and is frequently associated with underlying adenocarcinoma. A panel of immunomarkers (CK7, CK20, CDX2, GCDFP-15) plus mucin stain not only can help with diagnoses but also predict the presence of underlying malignancies. Long-term follow-up after local excision is required to monitor the disease recurrence and metastasis.

PPD must be differentiated from other squamous intraepithelial lesions, including squamous cell carcinoma and melanoma. Squamous lesions are positive for p63, p40 and high molecular weight keratin CK5/6. They are usually associated with Human papillomavirus (HPV) infection and p16 overexpression [19, 20]. Melanocytic lesions are usually positive for S100, SOX10, HMB45 and MelanA, but negative for cytokeratins [21], although rare cases can lose melanocytic markers and gain aberrant expression of cytokeratins [22]. Atypical regenerative basal keratinocytes sometimes can be mistaken as Paget’s cells; however, basal keratinocytes have intercellular bridges and are positive for squamous markers.

In contrast to what have been reported before [23–25], we found that CK7/CK20 were variably expressed in both primary and secondary PPD, yet GCDFP-15 was only expressed in primary PPD while CDX2 was only positive in secondary PPD, indicating CDX2 and GCDFP-15 are most reliable markers to distinguish these two subtypes of PPD. Our results suggest that the presence of CDX2+/GCDFP-15- PPD should prompt a careful search for primary adenocarcinoma in the lower gastrointestinal tract as the underlying adenocarcinoma determines the outcome of the patient. GATA3 is a very sensitive marker for primary genital and vulvar extramammary Paget’s disease [26, 27]. It is also positive in primary PPD, although may not be used to differentiate from secondary PPD. In our study cohort, 8 cases had a concurrent invasion adenocarcinoma, while 1 case had only tubular adenoma with high-grade dysplasia. Immunohistochemical stains confirmed that the Paget’s cells shared the same immunoprofile as the adenocarcinoma component, suggesting pagetoid spread from the underlying carcinoma cells. Only one case of PPD with concurrent tubular adenoma was reported before [11]; however, in such a case close follow-up is recommended to exclude occult invasive carcinoma.

In our case series, more than two thirds of PPD are classified as secondary, slightly higher than previously reported [5, 6]. It seems that the underlying malignancy dominates the clinical course and prognosis. Treatment and management should be primarily directed towards the underlying invasive carcinoma in addition to addressing the anal skin lesion by a variety of modalities. Primary PPD appears to be more indolent and patients usually die of other unrelated conditions. Wide local excision of skin and subcutaneous tissue in the perianal region is generally recommended for the treatment of the non-invasive form of PPD [28–30]. Radiation therapy is considered a treatment strategy in patients who were poor surgical candidates [31]. Other non-surgical treatments such as 5-fluorouracil or topical imiquimod have been used either in non-invasive or recurrent PPD [18, 32, 33]. photodynamic therapy with topically applied 5-aminolevulinic acid has been reported to treat non-invasive PPD and achieved complete cure without recurrence [34]. Although only a few cases were tested in our cohort, targeted therapy may be offered in situations if tumor cells are MMR deficient or have targetable genetic mutations.

In summary, we presented a multicentric study on PPD to characterize its clinicopathologic and immunophenotypic features. PPD is frequently associated with underlying adenocarcinoma, sometimes even a precursor lesion such as tubular adenoma. Mucinous and neuroendocrine features are not uncommon in the underlying malignancies and the Paget’s cells frequently demonstrate signet ring cell or mucinous features. CDX2 and GCDFP-15 proved to be the best distinguishing markers for primary vs. secondary PPD. Such a distinction is prognostically significant as the outcome in patients with secondary PPD is primarily dependent upon the invasive adenocarcinoma. Future studies may be warranted to explore the molecular signatures of Paget’s cells, as well as the mechanisms of pathogenesis, either de novo, or secondary.

## Data Availability

The datasets generated and/or analyzed in this study are available from the corresponding author upon reasonable request.

## Abbreviations

H&E: Hematoxylin and eosin
HPV: Human papillomavirus
MMR: Mismatch repair proteins
PPD: Perianal Paget’s disease
